# Patterns of Genetic Structure and Linkage Disequilibrium in a Large Collection of Pea Germplasm

**DOI:** 10.1534/g3.117.043471

**Published:** 2017-06-13

**Authors:** Mathieu Siol, Françoise Jacquin, Marianne Chabert-Martinello, Petr Smýkal, Marie-Christine Le Paslier, Grégoire Aubert, Judith Burstin

**Affiliations:** * Institut National de la Recherche Agronomique (INRA), Unité Mixte de Recherche (UMR) 1347, Agroécologie, 21065 Dijon, France; † Palacky University, Faculty of Science, Department of Botany, Holice, 783 71 Olomouc, Czech Republic; ‡ INRA, US 1279 Etude du Polymorphisme des Génomes Végétaux (EPGV), Centre de Recherche Ile-de-France-Versailles-Grignon, Commissariat à l’énergie atomique (CEA)-Institut de Génomique, Centre national de génotypage (CNG), Université Paris-Saclay, 91000 Evry, France

**Keywords:** genetic diversity, linkage disequilibrium, F_ST_, *Pisum sativum*

## Abstract

Pea (*Pisum sativum*, L.) is a major pulse crop used both for animal and human alimentation. Owing to its association with nitrogen-fixing bacteria, it is also a valuable component for low-input cropping systems. To evaluate the genetic diversity and the scale of linkage disequilibrium (LD) decay in pea, we genotyped a collection of 917 accessions, gathering elite cultivars, landraces, and wild relatives using an array of ∼13,000 single nucleotide polymorphisms (SNP). Genetic diversity is broadly distributed across three groups corresponding to wild/landraces peas, winter types, and spring types. At a finer subdivision level, genetic groups relate to local breeding programs and type usage. LD decreases steeply as genetic distance increases. When considering subsets of the data, LD values can be higher, even if the steep decay remains. We looked for genomic regions exhibiting high level of differentiation between wild/landraces, winter, and spring pea, respectively. Two regions on linkage groups 5 and 6 containing 33 SNPs exhibit stronger differentiation between winter and spring peas than would be expected under neutrality. Interestingly, QTL for resistance to cold acclimation and frost resistance have been identified previously in the same regions.

In crops, patterns of genetic diversity and the extent of linkage disequilibrium (LD) often result from a complex evolutionary history, including domestication bottlenecks, selection of favorable alleles, secondary admixture, or introgression of genetic material from wild relatives into cultivars. Studying these processes in crop species has proved of tremendous interest to evolutionary geneticists and breeders alike (Vigouroux *et al.* 2002; Ross-Ibarra *et al.* 2007). The domestication process of pea (*Pisum sativum*, L.), although as ancient as 10,000 yr (Zohary and Hopf 2000) is still a matter of debate. A few studies, however, have investigated genetic diversity at the species level, and results tend to indicate a surprisingly high level of genetic diversity in the cultivated gene pool given its highly inbreeding reproductive system (Baranger *et al.* 2004; Jing *et al.* 2010; Burstin *et al.* 2015). This could result from a weak bottleneck at domestication, important diversification after diffusion in Asia, Africa, and Europe, and/or gene flow between wild and cultivated material, none of these being mutually exclusive.

Concerns related to rapid human-induced climatic changes and increasing food demand owing to population growth have rekindled an interest in better characterization of the extant genetic and phenotypic diversity in cultivated plants. There is a wide spectrum of phenotypic diversity in pea, relating to varied agricultural practices and characteristics of the cultivated material (sowing date, usage, etc.). The genomic regions underlying many of these key adaptations are of particular interest, yet only a few have been roughly identified, mainly using QTL mapping approaches. The advent of high-throughput genotyping technologies in pea enables one to look for the footprints of selection using a different approach, scanning the genome to identify unusual regions in terms of diversity, LD, allelic differentiation and other genomic characteristics. These so-called genome scans have been applied in other crops, and represent an interesting complement to more targeted QTL, or association mapping, approaches (Schmutz *et al.* 2013; Siol *et al.* 2010).

Understanding the patterns of LD in germplasm and breeding collections is also of great relevance for applied genetic studies looking for genomic regions or QTL underlying traits of agronomic interest. In particular, the scale at which LD decays is one of the main factors to consider when evaluating the density of markers necessary to achieve sufficient power in association mapping or genomic selection approaches. This is particularly important in species such as pea, with a very large genome (∼4.45 Gb). While genetic diversity and genetic structure have been investigated on various collections in pea, examination of LD patterns have been conspicuously scarce to date (Jing *et al.* 2007). With the recent increased availability of markers across the genome (Tayeh *et al.* 2015a), it is now possible to assess how genetic diversity is distributed at the species level, as well as LD patterns with increased power.

The present study aimed to (i) describe the diversity patterns in a collection of germplasm representing elite cultivars, landraces, and wild peas; (ii) assess LD decay in different panels, and try to correlate it with the genetic characteristics of the panels; and (iii) identify genomic regions that might have been under selection either during domestication or postdomestication.

## Materials and Methods

### Plant material

**Figure 1:**
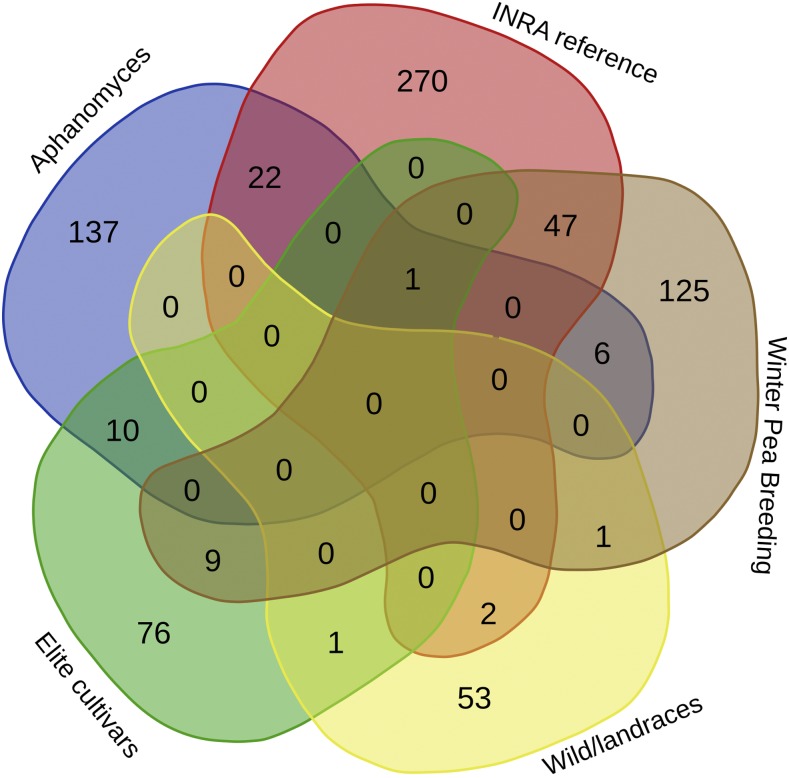
Venn diagram showing the number of accessions of each of the five panels (Elite cultivar, INRA reference collection, Winter Pea Breeding, Wild/landrace, and Aphanomyces Breeding). Figure obtained using http://bioinformatics.psb.ugent.be/webtools/Venn/.

A total of 917 *Pisum* accessions was selected from various collections. Overall, the goal was to represent the full spectrum of genetic variability from wild peas (*Pisum fulvum* and *P. sativum elatius*) and old landraces to modern elite cultivars, and also spanning the usage spectrum (winter *vs.* spring sowing types, fodder, field, and garden peas). Other genetic stocks and reference lines were included, in particular accessions exhibiting some resistance to *Aphanomyces euteiches* (Desgroux *et al.* 2016). A number of subsets have been defined in the current total sample: (i) the first [hereafter called INRA (Institut National de la Recherche Agronomique) reference collection] contains 342 genotypes from the 372-accession pea genetic resource collection described in Burstin *et al.* (2015), and is reasonably representative of the whole sample; (ii) the second consists of 189 genotypes representing the history of winter pea breeding programs (called Winter pea breeding) gathered by A. Baranger (UMR IGEPP Rennes); (iii) a set of 176 genotypes derived from crosses between garden pea from the United States, and pea showing good levels of resistance to *A. euteiches* (Aphanomyces breeding) provided by M.L. Pilet-Nayel (UMR IGEPP Rennes); (iv) a set (called Elite cultivars) of 97 elite cultivars from Europe, Canada. and the US; and (v) a set of 57 genotypes representing mostly wild material and old landraces from the Middle-East (called Wild/landrace) provided by P. Smýkal (University of Olomouć, Czech Republic). Figure 1 shows the overlap between these subsets. Even though the global sample is heterogeneous in nature, we aimed primarily at identifying patterns of genetic similarity and redundancy within the sample. This was done using a high marker density and a method that is essentially indifferent regarding the particulars of the genetic model that gave rise to the data (see *Genetic structure and MAF distribution*).

### Genotyping

The total sample was genotyped using the newly developed custom Infinium BeadChip (Illumina, San Diego, CA) Genopea 13.2K SNP Array as described in Tayeh *et al.* (2015a). This SNP array has been defined so as to include a maximum of mapped SNP. This generated a bias toward SNPs that were polymorphic among parents of *P. sativum* recombinant inbred line populations (Tayeh *et al.* 2015a). Data were analyzed using the Genotyping Module v1.9.4 of Illumina’s GenomeStudio software version 2011.1 (http://support.illumina.com/array/array_software/genomestudio.ilmn). When necessary, GenoPlots were edited manually so that three genotype clusters AA, AB, and BB could be obtained. Filters were applied to discard SNPs with missing rate ≥0.2, and/or heterozygosity ≥0.05, in further analyses.

For genetic structure analyses, SNPs with minor allele frequencies <0.02 were discarded, while, for LD analyses, the minor allele frequency (MAF) threshold was 0.05. Only the very best markers were kept (11,142), essentially those with a high-quality GenoPlots score and a high mapping quality on individual and consensus maps of biparental populations as described in Tayeh *et al.* (2015a).

### Genetic structure and MAF distribution

To investigate the genetic structure of our material, we used two methods: the first, Discriminant Analysis on Principal components (DAPC), implemented in the Adegenet R package (Jombart *et al.* 2010), is nonparametric, since it does not rely on any assumption regarding the genetic make-up of the data. By contrast, the second (fastStructure) uses an approach similar to the widely used program Structure, but tailored to deal efficiently with dataset containing several tens of thousands of markers through a variational Bayesian framework (Raj *et al.* 2014). It postulates a population genetic model, as do various other popular approaches, such as STRUCTURE or InStruct (Pritchard *et al.* 2000; Gao *et al.* 2007). The comparison of the results obtained with these two methods allowed us to evaluate the impact of violating the assumptions made by fastStructure.

DAPC was run without prior knowledge of groups, and the optimal number of clusters was thus assessed through sequential k-means and model selection using the Bayesian Information Criterion. The number of principal components was determined to be 13 through maximization of the α-score (measuring the difference between the proportion of successful reassignment of the analysis and values obtained using random groups), and the number of discriminant axes was set to six for a proportion of explained variance of 35.7%.

FastStructure was run for a number of clusters (*K*) ranging from 1 to 20 with five replicates for each value of *K* and using the “simple prior” option (flat beta-prior over allele frequencies). To evaluate the repeatability across runs, and rule out for true multimodality (as opposed to cluster labels switching), we ran the program CLUMPP v.1.1.2 using the Greedy algorithm (Jakobsson and Rosenberg 2007). The putative optimal number of clusters was assessed from the likelihood profile and by confronting the results with the DAPC analysis, and admixture plots were obtained using a custom python script.

Using the same genotypic data, we also calculated a simple distance matrix between individuals by counting the number of different alleles over the total number of alleles, accounting for missing data. This distance matrix was used to create a hierarchical tree with the Ward clustering algorithm with the hclust function in *R*. The tree was edited and displayed using the Interactive Tree of Life website (http://itol.embl.de/). Finally, we also examined the MAF distribution overall, and in different sets identified through the structure analysis above (namely wild/landraces, spring peas, and winter peas), as well as the joint distribution of allele frequencies between pairs of such ensembles.

### LD and kinship comparisons

For all LD analyses, SNPs were ordered according to the consensus genetic map produced by Tayeh *et al.* (2015a). LD *r*^2^ values were calculated between SNPs located on the same linkage groups and plotted against pairwise genetic distance using custom python scripts and functions from an expanded version of the EggLib package (De Mita and Siol 2012). More specifically, the mean and quantiles of the distribution of *r*^2^ values were calculated and plotted, both for all the linkage groups pooled together and by linkage group. We also plotted the *r*^2^ values as a function of the physical distance between SNPs found on the same genomic scaffolds, and for which a physical distance could be calculated.

To evaluate the magnitude of the effect of sample structure on the value of LD values, we computed the *r*^2^*_V_* value (Mangin *et al.* 2012), which estimates the correlation in allele frequencies correcting for the effect of relatedness by using a kinship matrix with the R package LDcorSV. The kinship matrix was estimated as the cross-product of the genotype matrix (with genotypes centered and standardized) in *R*. A heatmap of raw and corrected *r*^2^ values was obtained using the LDheatmap package in *R*. To allow for comparison in the level of kinship between different subsets, submatrices were extracted from the global kinship matrix. Since the kinship calculated in this way is dependent on allele frequencies, we also computed the identity-by-state (IBS) as described in Rincent *et al.* (2012), which measures the genetic similarity without regard to allele frequencies.

### F_ST_ scan

Following our analysis of genetic structure, we wanted to investigate which regions of the genome were the most divergent between wild, winter, and spring peas to pinpoint potentially interesting candidate genes. To do so, we computed both F_ST_ and Jost’s D genome-wide on an SNP basis, and plotted the values obtained ordering them using the genetic map. F_ST_ measures the proportion of the variance in allele frequencies attributable to variation between populations (Charlesworth and Charlesworth 2010), and has a long history of being used as a proxy for the level of differentiation between populations in population genetics. It has been noted, however, that its value is constrained by the level of heterozygosity of the marker used, and other measures have been proposed, such as Jost (2008). This was implemented by custom Python scripts using the revised version of the EggLib Package (De Mita and Siol 2012). The same was done with Nei’s heterozygosity. To test more formally for outliers in the F_ST_ genome scan that could constitute good candidates for loci having undergone selection for local adaptation, we used the BayeScan software (version 2.1). The method use a Bayesian framework to estimate the posterior probability of a locus being under selection by contrasting two alternative model of divergence from a ancestral population, one with selection and one without (Foll and Gaggiotti 2008). Annotations for the candidate SNPs identified through this approach were obtained from the Pea RNA-Seq gene atlas portal (Alves-Carvalho *et al.* 2015).

### Data availability

The genotyping data, the consensus map and the python and *R* custom scripts to analyze the data are all available in Supplemental Material, File S1.

## Results

### Genetic structure

**Figure 2:**
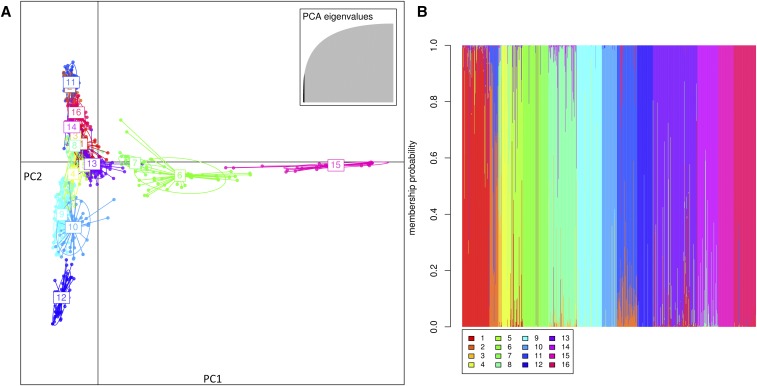
Genetic structure of the collection of 917 accessions of pea. (A) Scatterplot showing the first two principal components of the DAPC. Wild peas are grouped in clusters 6 and 15, winter sown peas are found in clusters 4, 9, 10, and 12, and the remaining clusters are spring sown peas. Note the position of the peculiar cluster 7, containing very original east Asian peas cultivated in tropical regions. The first axis nicely segregate wild *vs.* cultivated material, while the second axis spring and winter peas. (B) Membership probability plot of individuals.

A collection of 917 *Pisum* accessions representing a wide diversity was genotyped using the Genopea 13.2K SNP Array. From the initial 13,204 SNPs on the 13.2K chip, we retained information from 11,142 SNPs for genetic structure analyses. Following the analysis with DAPC, and, using the BIC profile, we chose to retain 16 clusters (see Figure 2A). These clusters tend to be consistent with information regarding the type of material and its use. Figure 2B shows the membership probabilities of each individual. When 16 clusters were considered, the results obtained with fastStructure were very congruent with the results from DAPC (results not shown). Three large ensembles emerge from this analysis: wild peas and landraces from the “Fertile crescent” and Asia (mainly Afghanistan, Nepal, and India, clusters 6 and 15), winter peas (clusters 4, 9, 10, and 12), and spring peas (all remaining clusters).

**Figure 3:**
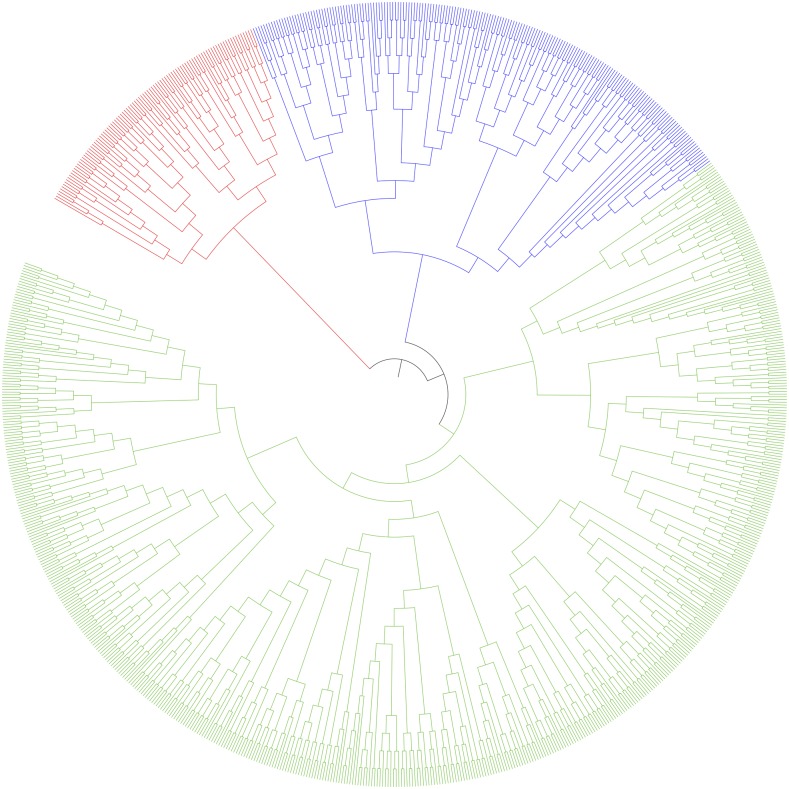
Global pattern of genetic differentiation. Distance tree based on a custom distance matrix measuring the number of shared alleles over the total number of alleles between two individuals. The tree was constructed using the Ward clustering algorithm. The distances are not represented to scale on the tree so as to make the dendrogram more readable. Red, wild/landrace; Blue, winter; Green, spring.

Reassuringly, there was an overall good correspondence between the groups as inferred from DAPC and the distance tree (Figure 3). Groups 4 and 9 mainly represented the history of winter pea selection, whereas groups 10 and 12 were composed of winter peas used mostly as fodder. Groups 14, 8, 3 and 16 were mostly dry seed peas. Two groups (2 and 11) were composed exclusively of genotypes from the Aphanomyces breeding subset (although other genotypes from the subset are not included in these groups). The constitution of two such groups is intriguing, and could be indicative of a technical artifact. One potential reason for the grouping of these accessions together, however, is that many of them are highly related recombinant inbred lines and not typical accessions in the classical sense (Desgroux *et al.* 2016). We thus decided to exclude these two groups from subsequent analyses involving spring peas. Groups 1, 5, and 13 were mostly garden peas, both recent and ancient varieties. A few fodder spring peas are also observed in group 13. Finally, group 7 is a very small, yet particular, group with only Far Eastern genotypes from China (see intermediate position between other cultivated peas and wild peas on the first axis of the discriminant analysis in Figure 2A).

Overall, the proportion of admixed individual, and the level of admixture, is lower when using the DAPC method than with fastStructure, as seen in other studies (Burstin *et al.* 2015; Roullier *et al.* 2013). Using the groups as defined by DAPC, the mean F_ST_ value (omitting groups 2 and 11) is 0.298.

As expected, the genotypes from the INRA reference subset are spread among the inferred groups, consistent with the fact that this collection has been built in order to best represent the diversity at the species level. There is, however, an imbalance in favor of group 1 (garden spring peas and fodder spring peas). The Aphanomyces breeding subset is strongly imbalanced, with most of its accessions found in either group 2, 11, or 16 (140 genotypes out of 176). As stressed before, groups 2 and 11 contain almost exclusively related genotypes from this subset. Accessions from the Winter pea breeding subset are scattered across different groups (with the exception of groups 2, 11, 15, and 16), but with a majority assigned to winter groups (4, 9, 10, and 12). Accessions from the Elite cultivar panel are predominantly dry seeds peas (group 8 and 14). Finally, and as expected, the accessions from the Wild/landrace panel are found almost exclusively in the two groups gathering the wild peas and old landraces of Middle-Eastern origin (groups 6 and 15). Overall, these groups are also found on the distance tree, although a few get mixed (4 and 9, 1 and 5, and 16, spread between different clusters of the tree).

### MAF distributions and kinship

**Figure 4:**
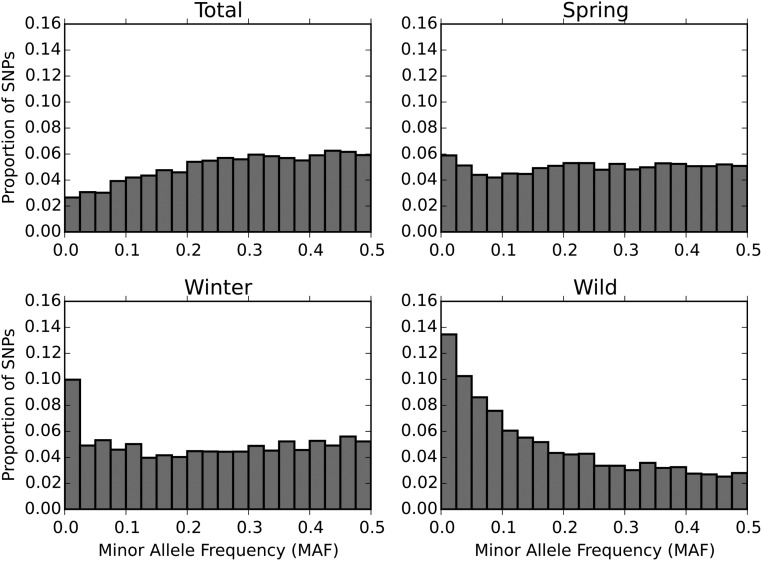
Histograms of MAF in the full dataset (total), spring, winter, and wild/landrace peas as identified by the DAPC analysis.

Figure 4 shows the MAF distribution for the total dataset, spring peas, winter peas, and wild/landraces peas—the three groups being defined through the previous analysis. The shape of the distribution on the total dataset results from the interplay of the allele frequencies in each subset. A striking feature of this distribution is the paucity of SNPs exhibiting very low minor allele frequency (Figure 4A). It is interesting to note the differences in MAF distribution in each subset; in particular, the wild peas MAF distribution has a very different shape than that of cultivated peas, with a lot more SNPs with rare alleles, and a relative paucity of intermediate frequency alleles.

**Figure 5:**
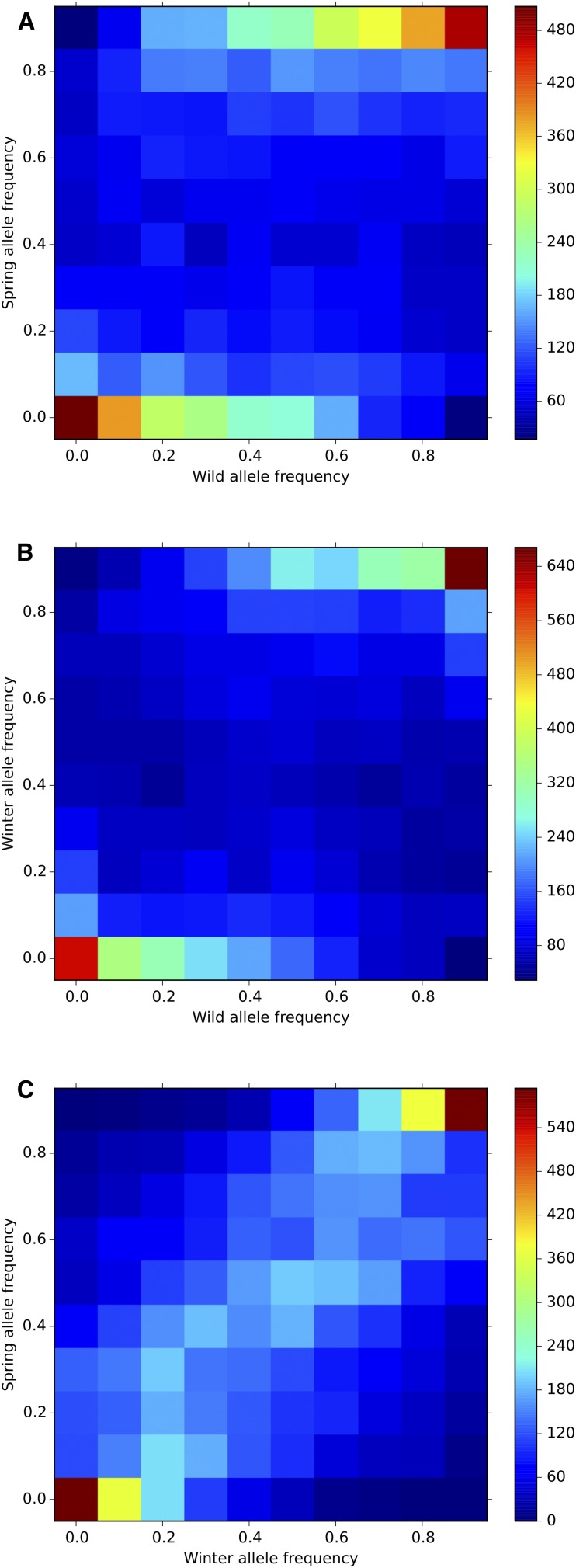
Joint allele frequency densities between wild and spring peas (A), wild/landrace and winter peas (B), and spring and winter peas (C). The color scale shows the minor allele frequency (MAF) counts (number of SNPs in that class of frequency, 10 equally spaced bins having been used to discretize the allele frequencies).

Figure 5 shows the joint allele distribution in the following pairs: winter–spring, winter–wild/landraces, and spring–wild/landraces. Allele frequencies are a lot more correlated between winter and spring peas than between either of them and wild/landraces peas; *i.e.*, alleles that are, say, at a low frequency in wild peas are less likely to be also at low frequency in cultivated peas. The distribution observed in wild peas is more akin to what is observed under a classic mutation/drift equilibrium model. There is probably a strong effect of the ascertainment scheme.

In our data, probably due to the ascertainment bias following the SNP selection for the Genopea 13.2K array (see *Materials and Methods*), wild peas appeared less polymorphic than cultivated peas with this set of markers. For example, the 17 accessions of *P. fulvum* are only polymorphic for 1764 SNPs (over the ∼11,000 used in this study). While many more markers were polymorphic in the accessions indicated as *P. s. elatius* (9172 markers), their relative allele frequencies differed drastically from those of cultivated pea.

**Figure 6:**
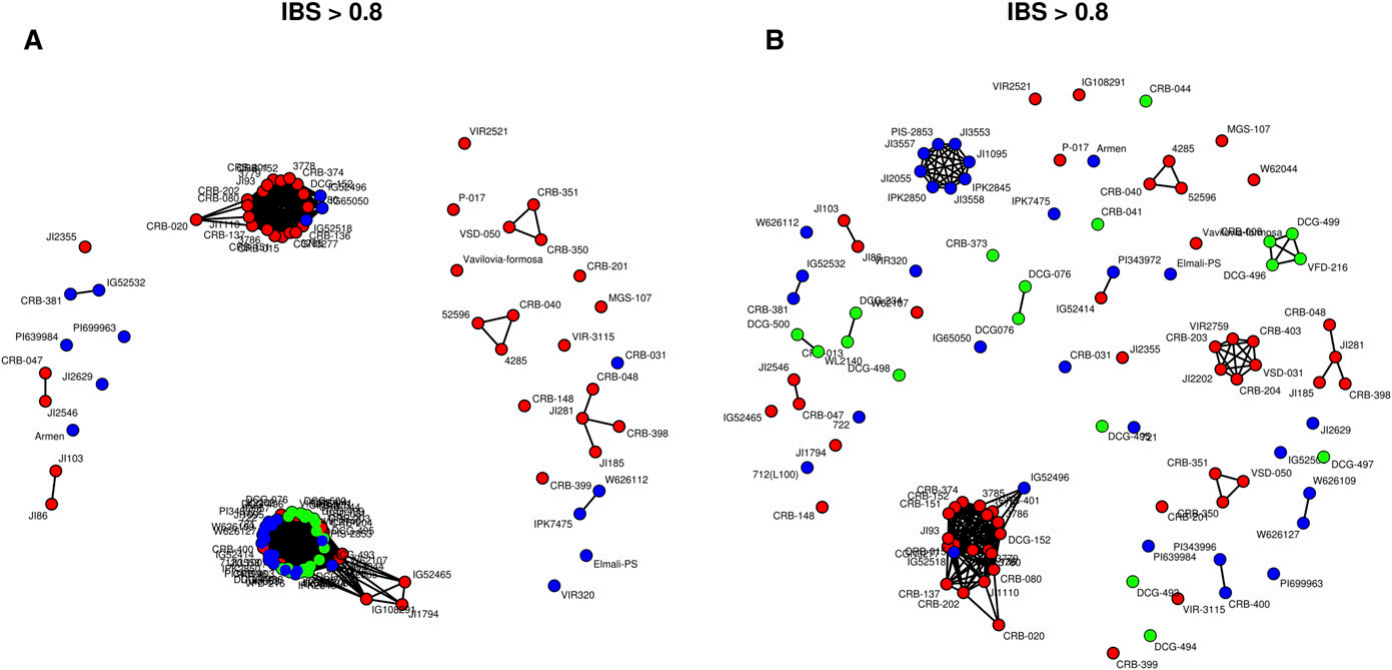
IBS networks of individuals belonging to the wild/landrace group. Only links showing an IBS >0.8 are shown on the network. (A) Network obtained with all the SNPs. (B) Network obtained using only SNPs polymorphic in *P. fulvum* (*n* = 1764). *P. fulvum* are indicated in green, *P. s. elatius* are indicated in blue on both networks.

Figure 6 shows an IBS network for the accessions belonging to the wild/landrace peas. Only links exhibiting an IBS >0.8 between any two accessions are shown. When using all SNPs, two very conspicuous groups of accessions showing high IBS are present. Interestingly, all *P. fulvum* accessions fall within the same group, while *P. s. elatius* are spread more evenly, indicating more genetic heterogeneity in the latter group. The second group gathers mainly *P. sativum* accessions from Afghanistan, India, and Nepal (roughly corresponding to cluster 6 in the DAPC analysis). Using the same accessions, but calculating IBS using only SNPs polymorphic in *P. fulvulm*, we observe that *P. fulvum* accessions are more spread out, while the group gathering Asian *sativum* is essentially unchanged.

### LD patterns

**Figure 7:**
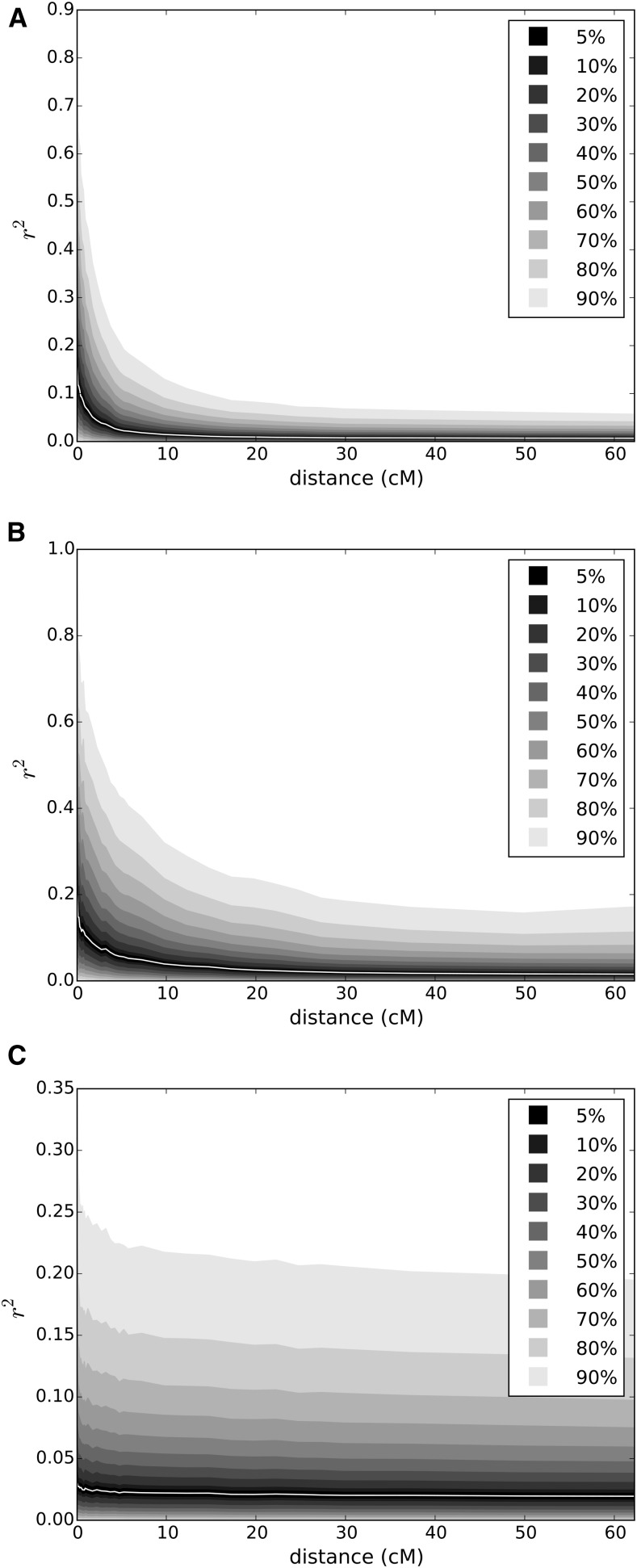
Patterns of intrachromosomal LD decay as a function of genetic distance over all linkage groups in (A) the spring (B) winter, and (C) wild/landrace groups. Each quantile from 5 to 95% around the median are represented. The genetic distances come from Tayeh *et al.* (2015a).

We investigated the patterns of LD decay in the genome using the classic *r*^2^ estimator as a function of the genetic distance. Considering the whole dataset, there is a steep decay in the LD values as the genetic distance increase; for example, the median value does not exceed 0.05 at a distance of 5 cM. Figure 7 shows that LD patterns are also strongly dependent on the sample considered; for example, while LD is low and decays steeply in spring peas, the trend is essentially similar in winter peas, but with qualitatively higher *r*^2^ values, and there is almost no LD in wild peas. This dependence upon the sample can also be observed by contrasting the panels available; for example, in the Elite cultivar subset, comprising mainly dry seeds pea from North America and Europe, the level of LD is substantially higher than in other subsets, although still decreasing rapidly (data not shown). This could be due to a history of higher levels of genetic drift, since the genetic basis in this sample is narrower.

**Figure 8:**
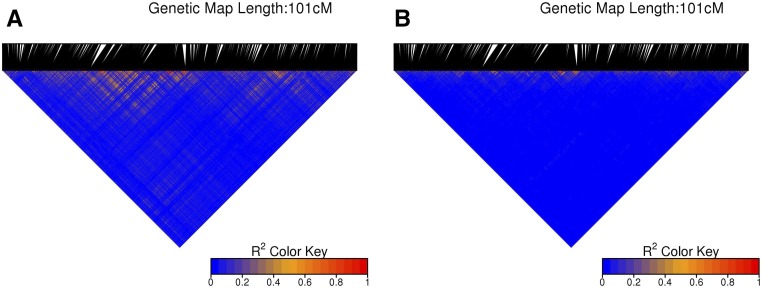
Heatmap of LD values both before (A) and after (B) correcting for kinship structure. Shown are the values for the sixth linkage group in the winter group. Kinship structure clearly accounts for some LD, especially at long range.

Population and/or kinship structure are known to generate long-range LD. To examine this in our dataset, we calculated the *r*^2^*_V_* estimator (Mangin *et al.* 2012), accounting for kinship. Results indicate that some of the LD observed in this dataset indeed come from the underlying genetic/kinship structure (see Figure 8, A and B for linkage group 6 in the winter group for an example).

### Fst scans

**Figure 9:**
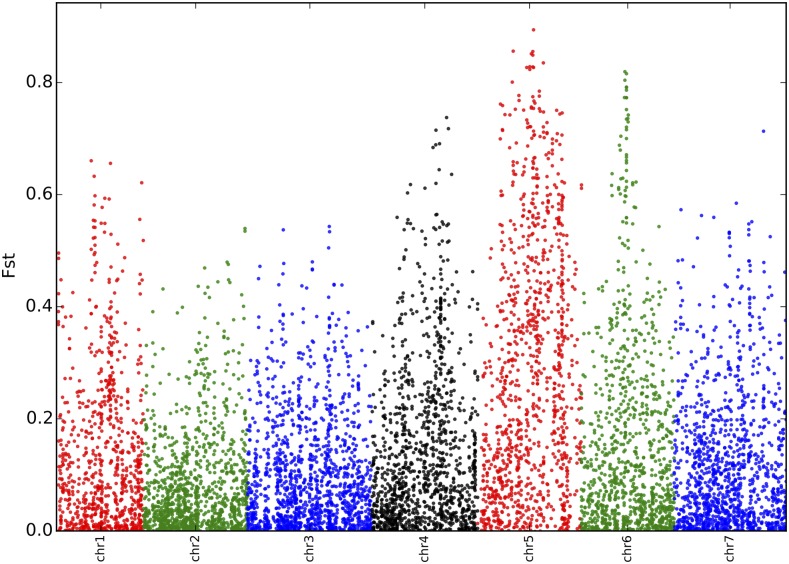
Manhattan plot of F_ST_ values across the genome contrasting spring *vs.* winter peas. SNPs are ordered according to Tayeh *et al.* (2015a).

A popular method to look for the signature of selection at the molecular level is to examine the level of differentiation along the genome. The underlying idea is that populations subjected to different environmental pressures might favor traits with different adaptive optima, and that these optima are attained by increasing the frequency of different alleles, respectively. As a result, regions of the genome exhibiting inordinately high levels of differentiation are good candidates for being involved in adaptation (to human needs in the case of a cultivated crop). A first simple way to visualize such regions is to plot the SNP-wise F_ST_ values on a Manhattan plot to look for obvious F_ST_ peaks. SNPs were ordered according to the consensus genetic map described in Tayeh *et al.* (2015a). Figure 9 shows a Manhattan plot of F_ST_ values between winter and spring peas, wild/landraces and winter peas, and wild/landraces and spring peas. Thereafter, we discuss only the results obtained for the winter/spring comparison, since no genomic regions could be identified that are particularly differentiated between wild and cultivated peas (either spring or winter types)—the F_ST_ being high across the whole genome. For the spring/winter comparison, a clear peak of differentiation could be observed in the middle of linkage group 6, while most of linkage group 5 showed high F_ST_ values. A more rigorous approach to detect significant outliers was undertaken, using the Bayesian framework described in Foll and Gaggiotti (2008). The method uses a model describing a number of subpopulations that evolved in isolation after splitting from an ancestral population. Each subpopulation may have experienced a varying degree of genetic drift, and the goal is then to determine the posterior probability that a particular locus has undergone selection. Using this method, with a threshold *q*-value set at 0.05 (meaning that, among the SNPs called significant, on average no more than 5% will be false positives), 33 SNPs were detected as exhibiting significantly higher levels of differentiation than expected under neutral divergence from an ancestral population. These SNPs (Table 1) were located only on linkage groups 5 and 6. A one-way ANOVA indicated significant differences between the mean F_ST_ values across linkage groups. While F_ST_ is often used as a proxy for population differentiation, its value is constrained by the heterozygosity at the marker. We calculated Jost’s D, which is specifically designed to measure differentiation in allele frequencies. The values were generally close to the values of F_ST_ as could be expected for SNPs with only two alleles per locus and a maximal value of heterozygosity of 0.5. Interestingly six kinases, along with three zinc-finger transcription factors, were found in these highly differentiated regions.

**Table 1:** List of SNPs detected as significant with BayeScan in a comparison between spring and winter peas

SNP	Linkage Group	Position (cM)	F_ST_	D	*q*-Value	Annotation
PsCam040463	5	37.8	0.8	0.74	0.019	C2H2 Zinc Finger
PsCam002784	5	38.8	0.85	0.77	0.003	Protein of unknown function DUF3741
PsCam030915	5	41.1	0.75	0.57	0.048	Putative nitrate transporter NRT1-3
PsCam000172	5	45	0.78	0.63	0.039	Oxoglutarate/iron-dependent oxygenase
PsCam051352	5	53.6	0.83	0.77	0.009	Photosystem II oxygen evolving complex protein PsbP
PsCam048068	5	56.9	0.83	0.79	0.006	Serine/threonine/tyrosine-protein kinase
PsCam050465	5	56.9	0.82	0.78	0.007	Putative aldose reductase-related protein
PsCam051635	5	58.6	0.85	0.79	0.003	Putative uncharacterized protein
PsCam040405	5	59.1	0.70	0.71	0.045	Phosphatidylethanolamine-binding protein PEBP
PsCam011361	5	60.2	0.85	0.80	0.004	Disease resistance?
PsCam012913	5	60.2	0.77	0.74	0.028	DNA cross-link repair protein
PsCam035714	5	60.2	0.86	0.81	0.001	LRR receptor-like serine/threonine-protein kinase
PsCam049838	5	60.2	0.83	0.75	0.008	Serine/threonine protein kinase-like protein
PsCam037956	5	61	0.83	0.80	0.007	BRO1 domain-containing protein BROX
PsCam039644	5	61	0.75	0.76	0.017	Zinc finger, C3HC4 RING-type
PsCam042222	5	61	0.89	0.82	0.001	Translation initiation factor
PsCam012545	5	61.4	0.77	0.77	0.014	—
PsCam031425	5	67.3	0.78	0.77	0.018	Bowman-Birk type proteinase inhibitor, Nodule expressed
PsCam048258	5	67.3	0.75	0.75	0.021	Probable serine/threonine-protein kinase
PsCam051833	5	67.3	0.78	0.78	0.010	Putative uncharacterized protein
PsCam058084	5	68.1	0.70	0.72	0.023	Zinc finger, LIM-type
PsCam042782	5	68.3	0.75	0.75	0.025	Flap endonuclease GEN-like
PsCam014128	5	71.7	0.83	0.79	0.005	LRR kinase
PsCam017406	5	71.9	0.75	0.74	0.032	—
PsCam038489	5	86.2	0.75	0.55	0.036	Dof Zinc finger protein
PsCam028287	6	46.9	0.77	0.65	0.043	Nuclear transcription factor Y subunit?
PsCam006662	6	47.4	0.80	0.66	0.015	YTH domain; evolutionarily conserved C-terminal region
PsCam022275	6	47.4	0.82	0.71	0.011	Putative retroelement polyprotein
PsCam004890	6	49.1	0.79	0.61	0.034	Heat shock protein Hsp20
PsCam023246	6	49.1	0.79	0.64	0.030	BAHD acyltransferase
PsCam037030	6	49.1	0.77	0.61	0.038	CBF-like protein CRT binding factor 1 CRT/DRE binding factor Drought responsive element binding protein 1
PsCam037082	6	49.1	0.79	0.69	0.027	SET domain
PsCam057485	6	49.1	0.82	0.63	0.013	Pyruvate kinase

Indicated is their position as well as F_ST_ and Jost’s D values and the associated *q*-value.

Finally, no obvious “dip” in genetic diversity (*H*_e_, Nei’s heterozygosity) could be noted along the genome in either winter peas or spring peas. Such dips have frequently been observed in domesticated species, and can be the result of selective sweeps around loci that have been particularly instrumental in the domestication process (Clark *et al.* 2004). Furthermore, the mean *H*_e_ value is lower in our wild peas than in both winter and spring peas (0.264 *vs.* 0.353 and 0.337). In order to make sure that this difference was not a byproduct of the difference in sample sizes, we generated 50 datasets for both the spring and winter peas containing the same number of individuals than the wild peas, and calculated the mean *H*_e_ over these 50 datasets. The strong difference remained, showing it was not due to differences in sample size, but more likely to the SNP selection process (see *Materials and Methods*).

## Discussion

Genetic diversity as a whole seems quite high in *P. sativum*, as has already been noted in previous studies (Baranger *et al.* 2004; Jing *et al.* 2007, 2010; Burstin *et al.* 2015). One of the primary goals of this study was to investigate the genetic diversity and structure patterns in a large collection comprising 917 accessions of field pea and a few relatives using a large array of SNP markers. Despite the heterogeneous nature of this sample, the overall picture provided by our study is very coherent with the biological characteristics of the material and its usage. At the highest level the dataset is divided in three groups: a group gathering all the wild peas/landraces, and two groups of cultivated peas, including all winter peas and most spring peas. These unequivocal groups identified through our study stem from actual genetic relationships between genotypes. The finer subdivision also makes good biological sense, especially when considering the cultivated material and its various usage type (food peas, feed peas, and fodder or garden peas), or the geographical origin, with, for example, clustering of some fodder winter peas from China (in cluster 7).

This type of structure according to cultivated type (winter/spring), end-use (feed, food, or fodder) has already been described in Baranger *et al.* (2004) and Burstin *et al.* (2015). Similarly, structure according to sowing types (winter/spring) and end-uses has been noted in wheat (Cavanagh *et al.* 2013) and barley (Comadran *et al.* 2012). The overall congruence between the nonparametric (DAPC) and parametric (fastStructure) methods tend to show that those assumptions likely to be violated by the constitution of our heterogeneous sample were not critical for the characterization of genetic structure.

In the present study, the panel used for SNP discovery was of modest size, comprising 16 accessions chosen to span the widest spectrum possible, so as to minimize ascertainment bias. However, the SNP selection criterion of being polymorphic in at least one *P. sativum* mapping population has introduced a bias in the survey of the diversity of wild accessions where the number of polymorphic markers was lower and the level of resolution achieved poorer. A resampling procedure ensured that this effect was not a side-effect of a lower sample size. Yet, the IBS networks of individuals belonging to the wild/landrace group showed interesting patterns of groupings among this group. Using SNPs that are polymorphic among *P. fulvum* accessions allowed better visualization of this group’s diversity. Regardless of the true level of genetic diversity in the wild material, the high genetic diversity in the cultivated material could indicate a scenario involving a weak bottleneck at domestication, possibly complemented by the diversity of uses, and the contrasting environmental conditions experienced in pea-producing areas worldwide.

While detecting the genomic regions affected by selection during domestication using this dataset seems difficult, we found a clear signal of strong differentiation on linkage groups 5 and 6 between spring and winter peas, strongly suggesting an effect of postdomestication selection. Interestingly, a QTL involved in frost tolerance is known on linkage group 6, and has been mapped between 49 and 53 cM using a different mapping population and mainly SSR markers (Tayeh *et al.* 2013). Our distinct F_ST_ peak nicely coincides with the position of this QTL. In particular, one of the markers detected under selection is in common between our studies [NT6083 in Tayeh *et al.* (2013), corresponding to PsCam057485]. A very interesting candidate (PsCam03730) is located in a contig exhibiting great homology with the CBFs genes in *Medicago truncatula* and *M. falcata*. As in *Arabidopsis thaliana*, the CBF genes have been shown to produce key transcriptional activators for cold acclimation (Pennycooke *et al.* 2008). Another QTL for frost tolerance has been found on linkage group 5, and mapped between 67.7 and 82.8 cM (again using a different map, Klein *et al.* 2014). Looking at the functional annotations, one of the more interesting candidate SNP (PsCam040405) is located on a gene encoding a phosphatidyl ethanolamine-binding protein (PEBP), and is mapped at 59.1 cM on LG5 in the map used for our study. This gene has been shown to be homolog of the *A. thaliana FT* gene in pea, which promotes flowering under long days (Hecht *et al.* 2011). In narrow-leafed lupin, another legume species, the loss of vernalization requirement has been shown to be associated with a deletion in the promoter, and a derepressed expression of a *FT* homolog (Nelson *et al.* 2017). Interestingly, the gene VRN-H3 found in barley is also a homolog of *FT*, and has been shown to be involved in the adaptation to spring growth in cultivated barley (Comadran *et al.* 2012).

Our data revealed a steeply decaying LD as a function of genetic distance, with a median *r*^2^ value of <0.05 at ∼3 cM on the total dataset, when all linkage groups are pooled together. The trend is the same regardless of the subsample considered, even though the values of LD can differ significantly. For example, LD is higher in the Elite cultivar and the Aphanomyces Breeding panels than in other panels. These differences are to be expected, since *r*^2^ strongly depends on allele frequency (Gianola *et al.* 2013), and, depending on how we subsample the data, the allele frequencies might vary widely. There is, for example, almost no LD when considering wild peas. A deeper evolutionary history with more time for recombination to break down LD could explain this difference with the cultivated material, which has most probably experienced bottlenecks and selection. These two factors are known to influence patterns of LD, along with other forces such as mutation, population structure, and admixture. For comparison in barley, another predominantly selfing crop, LD (*r*^2^) was observed to drop below what they called a basal level (0.2) at a distance of ca. 10–15 cM (Pasam *et al.* 2012). Comparison between species using genetic distances is, however, fraught with difficulties.

The extent of LD is an important factor to consider in light of the increasing interest in applying genome-wide association methods and genomic selection to pea. Indeed, the chromosomal extent of LD dictates the density of markers necessary to achieve sufficient power to detect associations, and the accuracy with which loci will be mapped. A recent study in one of our laboratories, however, has applied genomic prediction methods to a collection of 339 accessions from the INRA reference collection with satisfying results (Tayeh *et al.* 2015b). This study showed that, provided that the markers used were relatively evenly distributed across the linkage groups, even a relatively reduced set of markers (down to <1000) was enough to obtain good prediction accuracy. The same trend has been noted in wheat and barley (Heffner *et al.* 2011; Lorenz *et al.* 2012).

**Figure 10:**
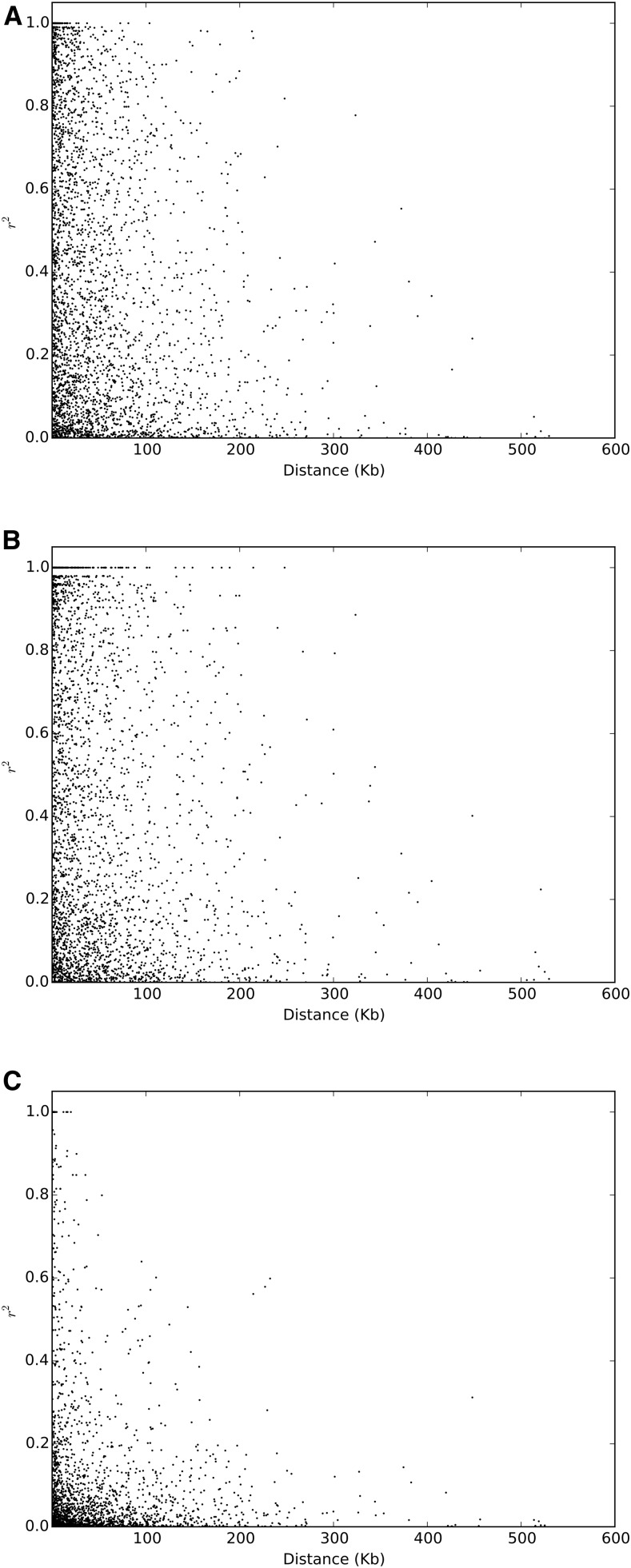
LD as a function of physical distance between SNPs. (A) spring peas, (B) winter peas, and (C) wild/landrace peas.

In the above, we plotted LD as a function of genetic distance, and it is therefore difficult to compare with other published reports of LD decay in crop species, where physical distances are available. The genome size of *P. sativum* is ∼4.45 Gb (Dolezel and Greilhuber 2010). With the consensus map used in this study, this indicates that 1 cM corresponds, on average, to a 5.6 Mb DNA chunks, this of course varying across the genome according to recombination rate. Using the set of SNPs located on identical scaffolds, we draw a first picture of the level of LD as a function of physical distance (see Figure 10). The plots obtained are necessarily sparser, since the number of SNP used was a lot smaller than for the previous analysis, but the trend is of a LD decaying on the scale of 200 kb in both winter and spring peas, and 100 kb in wild/landraces peas. Genome-wide LD decay rates have been estimated at ∼123 and ∼167 kb in 14 *indica* and *japonica* rice landraces (Huang *et al.* 2010), and the long range LD in cultivated rice is estimated at between 100 and 200 kb (Mather *et al.* 2007). This is often considered as a quite long-ranging LD, and has been linked to the self-fertilizing nature of rice, coupled with a small effective population size. Therefore, considering the genetic distance and physical distance can leave a different impression with our data. Indeed even though LD decays steeply with genetic distance, a centimorgan still spans a big physical distance in pea, and many genes can still be in substantial LD. However, we should wait for the full pseudomolecules to be available in order to reassess the level of LD decay using physical distances with more confidence and draw firmer conclusions.

In stark contrast with other well studied crops such as rice, barley, wheat, or maize, the domestication scenario particulars (timing and bottleneck intensity) in pea are not well known, and the taxonomy within the *Pisum* genus is not fully resolved, with opinions varying with regard to the number of species and their relations (Vershinin *et al.* 2003; Smýkal *et al.* 2012; Weeden 2007; Ladizinsky and Abbo 2016). Currently, the preferred hypothesis recognizes three species: *P. sativum* (including *elatius* which is thought of as the wild ancestor of cultivated peas), *P. fulvum*, and *P. abyssinicum*, which might have been domesticated independently (Maxted and Ambrose 2001).

A more in-depth investigation of the evolutionary history of the *Pisum* genus, its domestication, and subsequent breeding should probably use whole-genome resequencing data to alleviate the ascertainment bias issue. A more balanced sample with a larger diversity of wild and old landraces would also be useful. Once a workable domestication model identified, the systematic search for the footprints of the domestication at the molecular level should prove more fruitful.

## Supplementary Material

Supplemental material is available online at www.g3journal.org/lookup/suppl/doi:10.1534/g3.117.043471/-/DC1.
